# Virus-like Particles as Preventive and Therapeutic Cancer Vaccines

**DOI:** 10.3390/vaccines10020227

**Published:** 2022-02-02

**Authors:** Anna Lucia Tornesello, Maria Tagliamonte, Franco M. Buonaguro, Maria Lina Tornesello, Luigi Buonaguro

**Affiliations:** 1 Molecular Biology and Viral Oncology Unit, Istituto Nazionale Tumori IRCCS “Fondazione G. Pascale”, via Mariano Semmola, 80131 Napoli, Italy; f.buonaguro@istitutotumori.na.it (F.M.B.); m.tornesello@istitutotumori.na.it (M.L.T.); 2 Innovative Immunological Models, Istituto Nazionale Tumori IRCCS “Fondazione G. Pascale”, via Mariano Semmola, 80131 Napoli, Italy; m.tagliamonte@istitutotumori.na.it (M.T.); l.buonaguro@istitutotumori.na.it (L.B.)

**Keywords:** virus-like particles, VLPs, cancer vaccine, antigen delivery, human papillomavirus, HPV, hepatitis B, HBV, human herpesvirus type 8, HHV8, Epstein–Barr virus, EBV

## Abstract

Virus-like particles (VLPs) are self-assembled viral protein complexes that mimic the native virus structure without being infectious. VLPs, similarly to wild type viruses, are able to efficiently target and activate dendritic cells (DCs) triggering the B and T cell immunities. Therefore, VLPs hold great promise for the development of effective and affordable vaccines in infectious diseases and cancers. Vaccine formulations based on VLPs, compared to other nanoparticles, have the advantage of incorporating multiple antigens derived from different proteins. Moreover, such antigens can be functionalized by chemical modifications without affecting the structural conformation or the antigenicity. This review summarizes the current status of preventive and therapeutic VLP-based vaccines developed against human oncoviruses as well as cancers.

## 1. Introduction

The expression of viral proteins in non-human cells, their purification, and auto-assembling into hollow virus-like particles (VLPs) lacking viral genomes, represent an effective and safe strategy to mimic the native virus structure and morphology [1].

**Figure 1:**
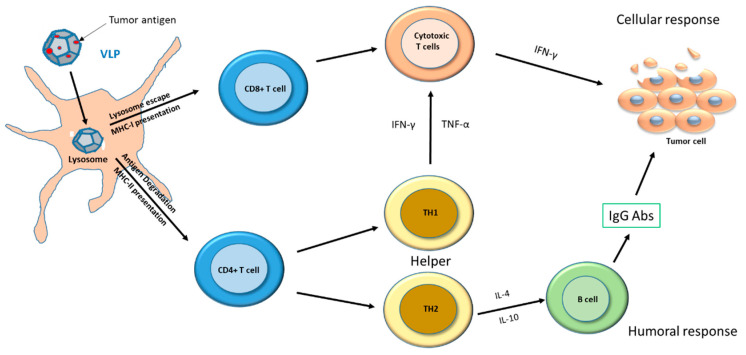
VLP-based vaccine: mechanism of action against tumor cells. VLPs are phagocytized and processed by DCs to present the tumor antigen on MHC-I and MHC-II for recognition by CD8^+^ and CD4^+^ T cells. CD4^+^ T cells differentiate into TH2 and TH1 cells that are involved in inflammatory response and in sustaining the activity of CD8^+^ T cells (cytotoxic T cells), respectively. CD8^+^ T cells exert cytotoxic activity on tumor cells.

Consequently, VLPs are able to efficiently interact with dendritic cells (DCs), the most potent antigen presenting cells, leading to viral antigen presentation on MHC class I and MHC class II molecules. Activated DCs migrate to lymph nodes to activate the antigen-specific CD4^+^ T helper as well as CD8^+^ cytotoxic T cells [2]. In particular, the CD8^+^ T cells become activated and exert their cytotoxic activity against chronically infected cells presenting the peptide MHC-I complex (Figure 1). 

CD4^+^ T helper cells are committed to differentiating two major subtypes, Th2 and Th1, driving adaptive immunity towards either a humoral or a cellular immune response, respectively [3,4]. Therefore, VLPs induce both humoral and cellular response and represent an effective vaccine strategy for preventive as well as therapeutic approaches [5].

It is well established that approximately 15–20% of all human cancers worldwide are associated and/or caused by human oncoviruses [6]. In particular, the International Agency for Research on Cancer (IARC) has recognized seven human viruses as class I carcinogenic agents directly involved in cancer development. They include human papilloma virus (HPV) types 16, 18, 31, 33, 35, 39, 45, 51, 52, 56, 58, and 59 (cancer of cervix, vulva, vagina, penis, anus, and head and neck); human T cell lymphotropic virus type I (HTLV-1) (adult T cell leukemia/lymphoma); human herpesvirus type 8 (HHV8) (Kaposi’s sarcoma and primary effusion lymphoma); Epstein–Barr virus (EBV) (Burkitt lymphoma, immunosuppression-related non-Hodgkin’s lymphoma, extranodal NK/T cell lymphoma (nasal type), Hodgkin’s lymphoma, and cancer of the nasopharynx); hepatitis C virus (HCV) (hepatocellular carcinoma and non-Hodgkin’s lymphoma); hepatitis B virus (HBV) (hepatocellular carcinoma), and Merkel cell polyomavirus (MCPyV) (Merkel cell carcinoma) [7]. Hence, the development of preventive vaccines that are able to induce protective antigen-specific immune responses and long-term immunological memory specific for oncoviruses, is the best strategy to prevent cancers caused by such viruses. In this respect, preventive vaccines based on VLPs have been developed for HBV and HPV and approved by international regulatory agencies for human use [8,9]. In particular, Cervarix, Gardasil, and Gardasil 9, which protect against the HPV virus, and Heplisav-B, which prevents the development of liver cancer caused by HBV infection [9].

On the contrary, different VLP-based therapeutic cancer vaccines have been evaluated in preclinical and clinical studies in different types of cancers, including melanoma, breast cancer, colorectal cancer, pancreatic cancer, and breast cancer [10,11,12]. Moreover, new technologies have allowed identifying patient-specific neoantigens that could be used to functionalize the surface of VLPs to develop personalized antitumor vaccines [13]. Nevertheless, despite the promising results, none have reached the phase III efficacy clinical stage [14]. In this manuscript, we describe the main developments in VLPs, as a platform for preventive and therapeutic vaccines against oncoviruses and cancers.

## 2. VLPs: Chemical and Biological Characteristics

**Figure 2:**
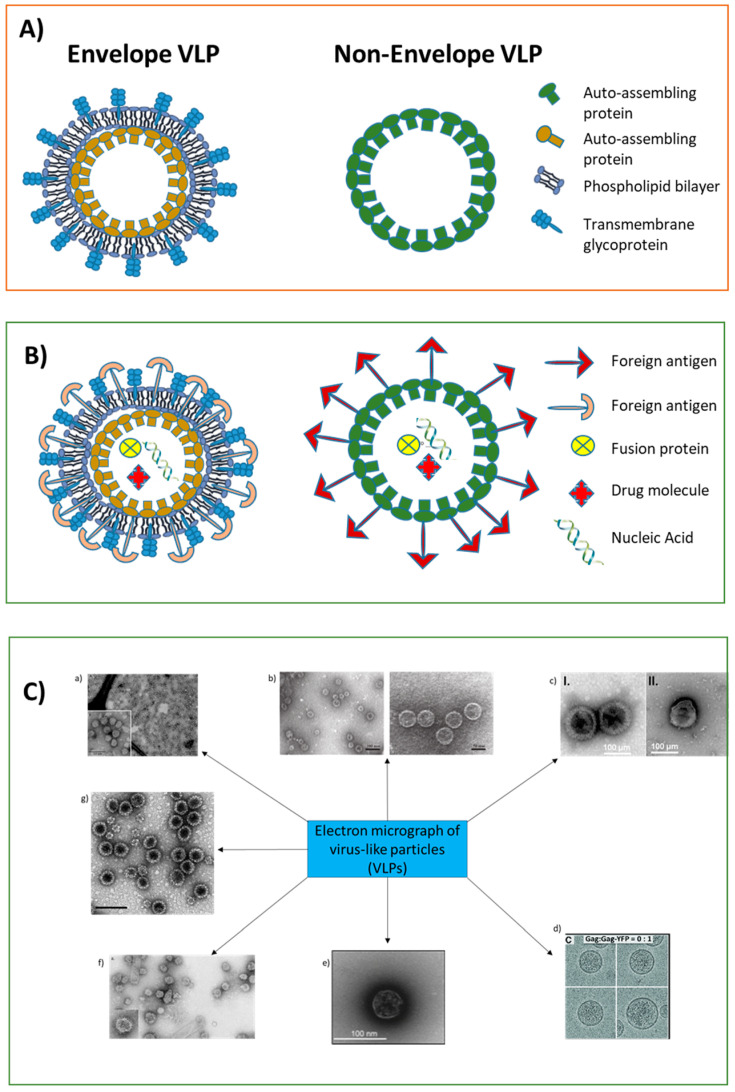
(**A**) Schematic structures of envelope (single layer, one protein) and non-envelope (single layer, one protein) VLPs. (**B**) Schematic structures of envelope and non-envelope VLPs as platforms for delivery of small molecules. (**C**) VLP electron microscope images. (a) HBsAg-VLPs [22]; (b) HPV-16 VLPs [23]; (c) I—KSHV virions; II—gpK8.1-based VLPs [24]; (d) HTLV-1 Gag VLPs [25]; (e) EBV-gp350/220-F VLPs [26]; (f) HCV-VLPs [27]; (g) MCPyV-VP1 VLPs [28].

Virus-like particles can be produced in different cells (e.g., yeast, bacteria), as well as in plants, and in insect or mammalian cell lines. The major differences between VLPs produced in mammalian cells and those produced in bacteria involve glycosylation, phosphorylation, or other post-translational modifications important for the immune response [15]. The production yield of VLPs is higher in bacteria and insect cells than in mammalian cells [15]. VLPs differ in size and geometry, which are crucial for the activation of T and B cells, and they elicit robust and long-lasting antibody responses, depending on the type of auto-assembling viral proteins [16]. The proteins forming VLPs are structured in icosahedron; they are rod-shaped, highly organized at up to three layers, and can contain a lipid outer envelope [17,18]. They have a diameter ranging from 20 to 100 nm; those below 40 nm were shown to have optimal uptake by dendritic cells [19,20]. Moreover, the internal cavity or the external surface of VLPs can be functionalized with specific ligands to deliver peptides, genes, and drugs to target tissues or to the immune system [11]. Small molecules are conjugated to the outer surface of VLPs via different structures. In particular, primary amines in the side chains of lysine, thiols in the side chain of cysteine, or phenols and carboxylic acids. Alternatively, unnatural amino acids modified with azide or alkyne groups are used to convey the molecules within the VLPs. Figure 2A,B shows a schematic illustration of VLPs used as a platform for the development of vaccines, and Figure 2C shows representative electron microscope images of VLPs generated against oncoviruses. Chimeric VLPs presenting the T cell epitopes of viral or cellular oncoproteins have been obtained by genetic fusion of a DNA-coding sequence in a specific position of the gene expressing the auto-assembling protein, in order to facilitate the presentation of the exogenous peptide on the outer surface of VLPs [21].

The chemical functionalization of VLPs is more expensive, but it prevents some troubles that can occur by genetic fusion, such as low protein yield or wrong folding of monomers, and it generally induces strong immune response with development of high titer of neutralizing antibodies [29]. The hydrophobicity scale and the appropriate length of the epitopes (15–20 amino acids) are important determinants for stable and structured VLPs [30]. The whole VLP production includes three steps of purification: (1) the cell lysis; (2) removal of large aggregates or cell debris; and (3) concentration. Different techniques are used to purify VLPs, such as sucrose or cesium chloride density gradient ultracentrifugation, high performance liquid chromatography (HPLC), size exclusion chromatography, or tangential flow filtration. For small scale preparations, the ultracentrifugation can be used for the purification or concentration method [31]. The characterization of nanoparticles is performed with electrospray ionization mass spectrometry time-of-flight (ESI-MS-TOF) or gas-phase electrophoresis [32,33]. The final steps of VLP preparation involve the sterile filtration and the vaccine formulation. Generally, VLPs are chemically stable in a broad range of temperatures and pH; however, it is possible to improve their stability by introducing disulfide bonds in the protein structure [34]. 

The immune response stimulated by VLPs is the result of their interactions with various components of the immune system. First, VLPs are internalized by DCs, then they are processed for the antigen presentation mediated by MHC-class I and MHC-class II molecules. Indeed, VLPs enter the pathway defined as “cross-presentation” inducing both helper CD4^+^ (MHC-class II) and cytotoxic CD8^+^ T cells (MHC-class I) (Figure 1) [35]. In particular, T helper cells can be driven to differentiate in either Th1 or Th2 subtypes able to sustain both arms of the adaptive immune response. Such an effect is extremely relevant for their applications in both preventive and therapeutic cancer vaccines. 

Both classes of molecules stimulate T cells, important for anticancer immunity, by presenting short epitopes on the cell surface in order to be recognized by (and within) the lymph node microenvironment (Figure 1). The epitopes binding the MHC-class I are generated from proteins derived from intracellular pathogens, while those binding the MHC-class II are generated from extracellular proteins upon internalization [36]. In addition, the latter can be presented by MHC-class I via a pathway [37]. CD8^+^ T cells differentiated into effector CTLs have the ability to lyse cancer cells [38,39]. Two major subtypes of effector CD4^+^ T cells have the potential to cause damage to the tumor microenvironment, such as Th1 cells, which produce the pro-inflammatory cytokines IFN-γ and TNF-α, and Th2 cells, which produce IL-4 and IL-10. 

Therapeutic VLP-based vaccines are designed to stimulate the adaptive immune system against specific tumor antigens in order to induce tumor regression [40]. Antibodies against tumor antigens expressed on cancer cells were also shown to cause cell death through different mechanisms, such as apoptosis, direct lysis, and cellular cytotoxicity [41]. VLPs also have the ability to stimulate B cell expansion and their differentiation to plasma cells producing IgG antibodies against specific antigens (Figure 1) [42].

## 3. VLPs Vaccine for Cancer Prevention

In the last 40 years, VLPs have been generated as preventive vaccine strategies for several viruses involved in the pathogenesis of human cancers, ultimately resulting in preventing cancer development. Of these, only two have received approval for human use by regulatory agencies, namely HBV and HPV VLPs. All other VLP models for the remaining oncogenic viruses are still in preclinical evaluation stage [43].

### 3.1. VLPs in the Prevention of Cancer Caused by Hepatitis B Virus (HBV)

HBV is an hepatotropic virus associated with chronic inflammation, progressive fibrosis, cirrhosis, and hepatocellular carcinoma [44]. The first VLP-based vaccine was developed for the hepatitis B virus (HBV) [45]. By the end of 1960, Dr. Baruch Blumberg unexpectedly discovered the new Australia antigen in sera of patients affected by acute and chronic hepatitis B, and he evidenced, for the first time, the formation of VLPs [46]. Later, the morphological characterization of the virus was studied with an electron microscope [47,48,49], and the assembly of HBV VLPs in the yeast expression system was achieved for the first time in the early 1980s [45,50]. The availability of HBV vaccines for all age groups represents the best health strategy to prevent HBV infection and related diseases. It is a small, enveloped virus of the Hepadnaviridae family, which is characterized by a circular and partial double-stranded DNA genome. The seven main proteins encoded by HBV include the structural moieties core, pre-core, small S, middle S, large S, polymerase, and the regulatory factor (HBx). VLP-based vaccines against HBV, in general, contain the small surface antigen S (HBsAgS) and the aluminum hydroxide adjuvant. Several types of anti-HBV vaccines have been developed throughout the years (Table 1) [51]. The first vaccine against HBV was the Heptavax-B, derived from HBsAg particles purified from the blood of patients infected with HBV; it was licensed in the United States in 1981 [52]. Second-generation HBV vaccines were genetically engineered VLPs developed by Merck and GlaxoSmithKline, by the stable expression of HBsAg in *Saccharomyces cerevisiae* and auto-assembling of the protein in 20 nm particles characterized by octahedral symmetric structures. The latter are safer and more immunogenic than blood-derived particles and are the most widely used today. The most recent vaccine formulation against HBV is the Sci-B-Vac, containing the S, pre-S1, and pre-S2 antigens, which are produced in mammalian Chinese hamster ovary (CHO) cells [53]. The production of Sci-B-Vac in CHO cells has the advantage of containing a combination of glycosylated and non-glycosylated HBsAg, with higher immunogenicity than those produced in yeast, which do not support any protein glycosylation. The Sci-B-Vac has been approved for use in Israel and East Asia in 2017 and more recently in 14 other countries [54]. The Heplisav-B is a new HBV vaccine made of HBsAg self-assembled VLPs, similarly to other third-generation vaccines, which contain the CpG sequence 1018 as adjuvant. It is already approved for use in adults in the United States and it is expected to be a next-generation vaccine used against HBV infection [51]. New strategies in the development of next-generation anti-HBV vaccines are represented by hyperglycosylated VLPs formed by different variants of HBsAgS subunits, tagged with an N-terminal FLAG epitope, such as T116N-HBsAgS (hyper-glycosylation), N146Q-ΔHBsAgS (non- glycosylation), or wild type HBsAgS. These are produced in HEK 293F cells grown in animal origin-free, protein-free mediums (FreeStyle medium) [55]. The hyperglycosylated VLPs administered with adjuvant aluminum hydroxide in BALB/c mice were shown to induce a stronger immune response compared to wild type VLPs [55]. Lastly, new methods to improve VLP production and safety have been developed, such as those based on *Pichia pastoris* cell-free synthesis of HBV proteins [56], showing comparable characteristics to those that the VLPs previously produced in yeast and mammalian cells [22,56].

### 3.2. VLPs in the Prevention of Cancer Caused by HPV

Human papillomaviruses are non-enveloped DNA viruses representing the most common genital viral contagions. The majority of HPV infections are usually cleared in a variable period of time, ranging from a few months up to 2 years, without any intervention. However, a small percentage of infections with high-risk HPV genotypes become persistent and can cause cancer at different body sites, such as the cervix, anus, vulva, vagina, penis, or oropharynx [57].

VLPs for HPV were initially developed in the early 1990s, expressing viral proteins from the outer layer of the HPV virus [58]. Since then, important developments have been made throughout the years on HPV structure, such as the achievement of the major capsid protein *in vitro* and the development of new models to study the assembly and structures of VLPs [23,59,60]. 

Two vaccines against HPV, derived from the viral capsid protein L1 and are named Cervarix and Gardasil, have been approved by the FDA for their ability to prevent HPV-infections and related cervical cancers [61]. Cervarix is a bivalent vaccine used against viral genotypes 16 and 18, while Gardasil was originally used against viral genotypes 6, 11, 16, and 18; since 2017, it has been a nonavalent vaccine used against HPV genotypes 6, 11, 16, 18, 31, 33, 45, 52, and 58 [62]. These vaccines are formulated with aluminum hydroxyphosphate sulfate, 3-O-desacyl-4’-monophosphoryl lipid (MPL) A, and aluminum hydroxide, respectively. Clinical trials are currently comparing the fractional doses of the two approved preventive vaccines (NCT04235257). The prophylactic HPV vaccine (Gardasil 9) is currently being evaluated, with researchers assessing the risk of HIL recurrence by 50% in previously unvaccinated individuals recently treated for anal or vulvar HSIL (NCT03051516). Both preventive HPV-VLP vaccines are highly immunogenic, but the high cost of production represents a significant limitation for their implementation in most less-developed countries [63]. The minor capsid protein L2 is important for the attachment and penetration of HPV particles into the epithelial cells, and it is considered as a good candidate for novel vaccine strategies against a broad spectrum of HPVs. Indeed, the L2 protein is highly conserved, giving the possibility of cross-protection against multiple HPV genotypes. However, the L2 alone is unable to form VLPs and a promising vaccine design is based on the multivalent display of HPV L2 peptides or epitopes on L1-based VLPs [64,65]. A further approach consists of the use of the thioredoxin (Trx) peptide, displaying L2 poly-peptides derived from L2 sequences of 5 to 11 diverse HPV genotypes. The Trx-L2 recombinant amino acid sequence was linked to the OVX313 heptamer, obtained from the complement inhibitor C4 binding protein (C4bp), for optimal presentation of the antigens to immune cells. Indeed, the OVX313 was shown to confer stronger immunogenicity to the Trx-L2 by acting as a strong inhibitor of the lectin pathways. Moreover, the Trx scaffold has been shown to induce strong humoral immunity and T- helper response. Notably, such vaccine formulation is able to induce a cross-neutralizing response against 14 high- and low-risk HPVs infecting the mucosa, as well as against several cutaneous HPVs [65]. A VLP vaccine, derived from the bacteriophage MS2 (16L2-MS2) exhibiting conserved L2 epitopes, has been tested in a preclinical study in female BALB/c mice. The 16L2-MS2 was highly immunogenic after a single dose and protected from infections against two heterologous pseudovirus (PsV) types, namely PsV31 and PsV45, while the Gardasil vaccine was partially protective against PsV31 and failed against the PsV45 challenge. The 16L2-MS2 vaccine was formulated in a dry powder that was stable at room temperature for seven months [66]. 

More recently, the structure of other oncoviruses have been characterized, including the polyomavirus major capsid protein VP1, expressed in *E. coli*, which assembled in virus-like particles assuming a pentameric structure [67,68]. Moreover, the morphological details of HTLV-1 particles are not well characterized, but different models have been used to produce HTLV-1-like particles, for example, the HTLV-1 Gag-only expression model system and MT-2 cells [69,70,71]. Similarly, the morphology and structure of HHV-8 and HBV are not well characterized due to the difficult cultivation in tissue culture or animals. Nevertheless, the comparison between the structure of the two viruses HHV-8 and HBV in virus-infected endothelial cells showed similar morphological features in the two nucleocapsids and capsomer organization [72,73]. Lastly, the structure of the HCV protein, which was expressed in insect cells and assembled into enveloped virus-like particles in large cytoplasmic cisternae, was well characterized [74]. Progress in the study of the structures of oncoviruses and the approaches to produce VLPs as platforms for immunological vaccines represents an opportunity to stop infections at early stages. Table 2 summarized the VLP vaccine for the prevention of cancer caused by oncoviruses.

### 3.3. VLP Vaccines against Human Herpesvirus Type 8 (HHV-8) to Prevent Kaposi Sarcoma Development

Human herpesvirus type 8 (HHV-8) or Kaposi sarcoma-associated herpes virus (KSHV), is a gamma herpesvirus that infects endothelial cells, epithelial cells, B-cells, and macrophages. The virus is able to promote B-cell proliferation, production of angiogenic cytokines, and VEGF expression, as well as impair the host immune system by escaping the cytotoxic immune response, the antigen presentation, and T cell activation. In particular, infected endothelial cells undergo extensive proliferation and neoplastic transformation. HHV-8 is the primary cause of classical and epidemic Kaposi’s sarcoma (KS), multicentric Castleman′s disease, primary effusion lymphoma, and a rare form of B-cell lymphoma [84,85]. The elevated secretions of pro-angiogenic factors, such as VEGF, IL-6, IL-8, angiopoietin, etc., are the major causes of HHV-8 oncogenic activities.

Only a few studies to date have been devoted toward developing a prophylactic vaccine against HHV-8, given that the incidence of HHV-8-associated tumors have been dramatically reduced by the efficacy of anti-retroviral treatment in HIV infection. Moreover, the lack of animal models for KS development represents a strong limitation in evaluating the VLP efficacy [86,87]. 

The HHV-8 glycoproteins gpK8.1, gB, and gH/gL are responsible for the virus attachment, to target cells and to elicit neutralizing antibodies. These glycoproteins have been individually fused to the Newcastle disease virus (NDV) capsid proteins and the transfection of chimeric HHV-8-NDV recombinant constructs in CHO cells yielded successful production of VLPs. The gpK8.1, gB, and gH/gL VLPs resembled the morphology of the native HHV-8 virus and were shown to bind the HMC-1 cell line, susceptible to HHV-8 infection [24].

Preclinical studies have shown that the VLPs were able to induce neutralizing antibodies in mice and that immunization with a combined formulation of gB and gH/gL VLPs induced the best neutralizing antibody response. Moreover, the administration of the gpK8.1 VLP vaccine with gpK8.1-gB, gpK8.1-gH/gL, or gpK8.1-gB-gH/gL VLPs induced an immune response that was similar to that obtained with UV-inactivated HHV-8 [88]. 

More recently, a multivalent VLP-based vaccine, incorporating four HHV-8 envelope glycoproteins, was produced by cloning a polycistronic sequence containing the gpK8.1, gB, gL, and gH glycoproteins interspersed with a picornavirus 2A self-cleaving peptide under the CMV promoter in a single plasmid [76]. Co-transfection of CHO or HEK-293 cells with plasmids expressing HHV-8 as well as NDV M and NP glycoproteins resulted in an efficient production of VLPs incorporating HHV-8 proteins. Immunization experiments in wild type New Zealand white rabbits with alum/MPL adjuvant HHV-8-VLPs or alum/MPL-adjuvant UV-inactivated HHV-8 showed that IgG antibodies were specific to all HHV-8 glycoproteins included in the VLPs. However, the titer of antibodies against HHV-8-VLPs decreased significantly after 70 days in comparison with that measured in rabbits immunized with UV-inactivated HHV-8. In addition, the IgGs purified from a pooled sera of rabbits immunized with HHV-8-VLPs were shown to neutralize the HHV-8 infection of epithelial, endothelial, fibroblast, and B cell lines [76].

### 3.4. VLP Vaccines to Prevent Cancers Caused by the Human T-Lymphotropic Virus 1 (HTLV-1)

The human T-lymphotropic virus type 1 (HTLV-1) was the first oncogenic retrovirus to be discovered in humans [89]. The HTLV-1 is associated with human diseases, such as adult T cell leukemia (ATL) and chronic inflammatory diseases of the central nervous system, defined as HTLV-1-associated myelopathy or tropical spastic paraparesis (HAM/TSP). The HTLV-1 is endemic in several geographic regions, including Japan, the Caribbean, Africa, South America, and the Melanesian islands. The Gag proteins have been used to generate produce HTLV-1-like particles [25,70]. Gag polyprotein is known to be the primary driver for virus particle assembly and release. HTLV-1-like particles were produced in MT-2 cells by expressing the HTLV-1 Gag protein, which was observed to auto-assemble into a spherical shape of 110 ± 32 nm, mimicking the morphology of immature HTLV-1 virions [70].

### 3.5. VLP Vaccines to Prevent Epstein–Barr Virus (EBV)-Related Cancers

Epstein–Barr virus (EBV), the first oncogenic virus identified in humans, is a gammaherpesvirus infecting epithelial and B cells. EBV principally causes mononucleosis and has been associated with diverse cancer types, such as lymphomas, gastric carcinoma, and nasopharyngeal carcinoma, diagnosed either in immune-competent or immune-compromised patients [90]. The development of a tumor associated with an EBV infection has a complex etiology and is heavily dependent on environmental factors and genetic susceptibilities in specific ethnic populations and geographic regions [91,92]. Much effort has been put in the development of a VLP-based prophylactic vaccine able to induce humoral as well as cell response against EBV in order to reduce the risk of developing EBV-associated malignancies [72]. A recombinant EBV genome, expressing auto-assembling proteins but not viral oncogenes, has been constructed to produce and release VLPs from 293-VII+ cells and via exosomes from HEK293 cells. The EBV-based VLPs were taken up by B cells, and the viral epitopes were presented to T cells in association with HLA molecules, providing effective immunity against EBV [72]. In addition, EBV-based VLPs not only reactivate EBV-specific CD4+ and CD8+ T cells from EBV-seropositive individuals but also prime broad-spectrum cellular and humoral immune responses in mice [72]. Another model of an EBV vaccine was obtained by fusing the EBV gp350/220 to the fusion F protein of the new disease virus (NDV) in order to the display gp350/220 ectodomain (ED) in VLP particulate form [26]. The EBV–VLPs were produced in Chinese hamster ovary (CHO) cells and the immunization of BALB/c mice elicited a durable and specific antibody response able to block EBV infection *in vitro* without the use of any adjuvant [93]. In addition, a polyvalent vaccine was developed by incorporating the glycoproteins gH/gL or gB, which are essential for the EBV entry, as well as the EBV nuclear antigen 1 (EBNA1) and latent membrane protein 2 (LMP2), expressed in all EBV-infected cells, on the surface of VLPs [77]. The gH/gL-EBNA1 and gB-LMP2 VLPs were efficiently produced in CHO cells and were shown to induced either high neutralizing antibody titers or EBV-specific T cell responses in BALB/c mice without the use of adjuvants. Therefore, these EBV–VLPs will be suitable as a preventive vaccine against EBV infection and as a therapeutic vaccine to treat EBV-associated cancers [77]. 

More recently, a pentavalent vaccine based on EBV glycoproteins gp350, gB, gp42, gH, and gL, exposed on the surface of VLPs, has been produced in CHO cells. This vaccine has been administered with the adjuvants aluminum hydroxide and monophosphoryl lipid A, eliciting a strong neutralizing antibody response in New Zealand white rabbits. This was shown to protect both B cells and epithelial cells from the EBV infection, better than soluble gp350 [78].

Finally, a promising strategy is represented by a multicomponent vaccine model, in which three gp350 peptides (P1 (aa 16–29), P2 (aa 142–161), and P3 (aa 282–301) were displayed on the surface of the HBV core antigen (HBc149) in different orders. All recombinant proteins were able to self-assemble in VLPs. Among these, the HBc-149-3A (P1P2P3) and HBc-149-3B (P1P3P2) VLPs induced high titers of neutralizing antibodies against gp350 in mice and blocked EBV infection in cell culture [79].

### 3.6. VLPs in the Prevention of Cancer Caused by Hepatitis C Virus (HCV)

Hepatitis C virus (HCV) is an enveloped, positive single strand RNA virus of the Flaviviridae family, characterized by a viral life cycle fully completed in the cytoplasm. The HCV RNA genome is translated in a single polyprotein of 3011 amino acids, cleaved to produce four structural (capsid protein C, envelope glycoproteins E1 and E2, protein P7), and six non-structural (NS2, NS3, NS4A, NS4B, NS5A, and NS5B) proteins [94,95,96]. VLPs for HCV have been generated by co-expression of the core, E1 and E2 proteins, in a baculovirus expression system. A heterologous prime-boost protocol, including the HCV VLPs and adenovirus expressing the HCV-core-E1-E2 proteins, resulted in the enhancement of both antibody and T cell responses against HCV proteins. The antibodies showed a neutralization effect, inhibiting the binding of the HCV JFH1 variant to the Huh-7 cells [27,81]. This expression system has also been used to produce a quadrivalent VLP-based vaccine against HCV genotypes 1a, 1b, 2a, and 3a, and has been shown to elicit antibodies and T cell responses against the HCV epitope cellular immune specific response [82]. Recently, such a quadrivalent vaccine was tested in the pig by intradermal administration, without the addition of adjuvants, eliciting long-lasting neutralizing antibodies against multiple HCV genotypes and strong T cell and granzyme B responses [83].

### 3.7. VLPs in the Prevention of Cancer Caused by Merkel Cell Polyomavirus (MCPyV)

Merkel cell polyomavirus (MCPyV) is a non-enveloped double-stranded circular DNA virus belonging to the α-polyomavirus genus, found in approximately 80% of Merkel cell tumors [97]. MCPyV-like VLPs were produced in the baculovirus system upon self-assembling of the VP1 protein and release into the culture medium [28]. The characterization of MCPyV VLPs showed that they are composed of 72 pentameric capsomeres arranged in a T = 7 icosahedral surface lattice of 48 nm in diameter [98]. The administration of MCPyV VLPs in mice induced a strong immune response, providing a good prerequisite for the development of a prophylactic vaccine to prevent MCC [99].

## 4. VLPs Vaccine for Cancer Therapy

Vaccines capable of boosting the immune response against cancer specific antigens represent a very promising therapeutic strategy for the targeted treatment of tumors. In this respect, VLPs represent an efficient platform for the presentation of tumor-associated antigens (TAAs). This can be achieved by two experimental approaches: (1) the outer surface of VLPs can be functionalized to bind the TAAs for the optimal presentation to immune cells (e.g., short linear or cyclic peptides, full proteins and non-protein targets, including glycans and haptens); (2) the coding sequence expressing the structural viral proteins forming the VLPs is modified to generate a chimeric protein presenting the desired TAA. In the first approach, the VLPs, the peptides/proteins, and linkers are synthesized separately and then combined by cross-linking reactions to produce complex vaccine formulations. In the second one, a single recombinant protein is expressed in the cell system, retaining the ability to auto-assemble in particles, and presenting the TAA, is expressed in prokaryotic or eukaryotic cells. Table 3 summarizes recent studies on VLP-based therapeutic cancer vaccines.

### 4.1. VLPs for Breast Cancer Therapy

Breast cancer is a heterogeneous tumor characterized by a high degree of histopathological and molecular diversity between and within tumors [109]. Due to breast cancer complexity and lack of specific treatments, there is an urgent need for new therapeutic strategies, such as cancer vaccines. The human epidermal growth factor receptor-2 (HER2) is overexpressed in about 30% of invasive breast cancers, comprising triple negative and HER-2+ breast cancers; thus, representing a promising cancer vaccine target. Recently, a new strategy to produce immunogenic VLPs has been pursued using the method termed the SpyCatcher–SpyTag system, which was developed to generate efficient protein ligation by isopeptide bonds [110]. The SpyCatcher–SpyTag system is derived from a modified domain (CnaB2) of Streptococcus pyogenes surface protein, FbaB (SpyCatcher), which binds a 13-amino-acid peptide (SpyTag) through a covalent isopeptide bond, linking a lysine side chain in SpyCatcher to an aspartate in SpyTag [110]. VLPs for a breast cancer vaccine have been produced by genetically fusing the SpyTag/SpyCatcher to the N- or C-terminus of the Acinetobacter phage AP205 capsid protein, which auto-assembled in stable VLPs expressing multiple SpyCatcher/SpyTags per each capsid protein and an irreversible conjugation of the HER-2 extracellular domain (subdomain I-IV) on the VLP [100,111]. These VLPs are characterized by high-densities of HER2 epitopes on their surfaces and in their ability to elicit strong anti-HER2 immune responses. The VLP formulation administered as a prophylactic vaccine reduced spontaneous development of mammary carcinomas by 50%–100% in human HER2 transgenic mice and inhibited the growth of HER2-positive tumors implanted in wild type mice [100]. A further cancer vaccine study demonstrated that the VLPs, obtained by human parvovirus B19 chimeric VP2 proteins displaying two epitopes of the insulin-like growth factor-1 receptor (IGF-1R), were effective against breast cancer in the animal model. Indeed, these VLPs were able to prevent and delay tumor growth when administered prior to the inoculation of 4T1 cells in female BALB/c mice [101]. The animals produced specific antibodies against the IGF-1R epitopes, suggesting their role in the anti-tumor effect, although the T cell responses were not significantly activated following stimulation by specific epitopes [101]. Another study evaluated the use of the bacteriophage MS2 VLPs, displaying the extracellular loop of the cysteine/glutamate antiporter xCT tumor-associated antigen, as a promising therapeutic target to inhibit tumor progression and metastasis formation in metastatic breast cancer (MBC) [102]. Indeed, a high percentage of invasive mammary ductal tumors, including triple negative breast cancer, show elevated levels of the xCT protein correlating with poor overall survival. The VLP-based xCT vaccine (AX09) was shown to be well tolerated, eliciting a robust antibody response against xCT and reduced metastasis formation in different breast cancer mouse models [102].

### 4.2. VLPs for Melanoma

Melanoma is the deadliest skin tumor characterized by the highest mutational burden of any cancer and numerous tumor-associated antigens, which can be used for the generation of effective therapeutic cancer vaccines [112]. A VLP strategy, based on the use of the cucumber-mosaic virus (CuMV) coat protein to display relevant epitopes for the induction of immune response, has been used to present the p33 epitope as a model antigen. The CuMVTT-VLPs-p33 were obtained by using bio-orthogonal Cu-free click chemistry and were formulated with the micron-sized microcrystalline tyrosine (MCT) adjuvant. The immunogenicity of such a formulation has been evaluated in an aggressive transplanted murine melanoma model. The results show that CuMVTT-VLPs can rapidly drain into the lymphatic system due to their small sizes (~30 nm), with a local “depot effect" when formulated with a micron-sized MCT adjuvant, resulting in prolonged exposure to the immune system [103]. 

A further model of the anti-melanoma vaccine is based on the use of cowpea mosaic virus-like particles (CPMV-VLPs), which showed promising results as an immune stimulating agent for in situ cancer vaccination [104]. Two VLP constructions have been developed: one is based on the complete CMPV virion, including the genomic RNA; the second is based on the empty CPMV virion [113]. The two formulations showed characteristic immunostimulatory effects, which are mainly due to the presence of RNA in the complete CPMV. Indeed, both types of VLPs induced similar cytokine profiles and activation of immune cells, but only those containing RNA were able to strongly activate the populations of antigen-presenting cells, such as tumor-infiltrating neutrophils and dendritic cells.

A dual-antigen vaccine strategy was developed, based on the hepatitis B virus core antigen presenting the OVA257−264 (SIINFEKL) and the gp100 (KVPRNQDWL) antigen peptides [105]. The chimeric HBc proteins expressed in *E. Coli* were able to assemble into VLPs and to slightly enhance the maturation of bone marrow-derived dendritic cells ex vivo. Notably, such VLPs induced a strong antigen-specific antitumor immunity in mice, and an innate immunity against tumors and metastasis originated from the subcutaneous implantation of B16-OVA melanoma cells [105].

### 4.3. VLPs for Pancreatic Cancer

Pancreatic cancer is an aggressive tumor with the worst survival rate of all cancers. The outcomes and survival benefits for pancreatic cancer patients—despite significant therapeutic developments—remain unsatisfactory, highlighting the urgent need for novel therapeutic strategies to fight this deadly disease. An immunotherapeutic approach for pancreatic cancer was developed, based on a murine Trop2 VLP approach. Indeed, the Trop2 glycoprotein is highly overexpressed on the cell surface of pancreatic cancer. Immunization of C57BL/6 mice with mTrop2 VLPs resulted in a significant reduction in tumor growth and broad activation of CD4(+) and CD8(+) T cells. In addition, the administration of Trop2 VLPs in combination with gemcitabine showed an improved survival of tumor-bearing mice [106]. A further immunotherapeutic target is the mesothelin (MSLN) glycoprotein that is specifically expressed on the surface of mesothelial cells and overexpressed in most pancreatic cancers. A chimeric murine MSLN-VLP has been shown to break the tolerance to the murine MSLN self-antigen, as well as reduce tumor growth and prolong overall survival in an orthotopic pancreatic cancer model [107]. The murine MSLN-VLP administration induced the enhancement of MSLN-specific CD8+ T cells and decreased FOXP3+ Treg cells, contributing to the suppression of tumor growth. A therapeutic vaccine is in the early stage trial study for the treatment of pancreatic cancer at stage IV, and other cancers, except melanoma (NCT04387071). The VLP CMP-001, containing a short fragment of DNA, was investigated in combination with the antibody INCAGN01949 due to its ability to activate the immune system and to recruit immune cells to the tumor. The injection of CMP-001 and INCAGN01949 directly into the tumor can slow down tumor growth by causing tumor cells to die. CMP-001, in combination with the monoclonal antibody pembrolizumab, is in phase II trial, to treat patients with melanoma (NCT04708418, NCT02680184).

### 4.4. VLPs for Cervical Cancer

HPV E6 and E7 oncoproteins are attractive targets for the development of therapeutic vaccines for HPV-associated cancers. Indeed, such proteins are tumor-specific antigens (TSAs) given that they are constitutively and specifically expressed in HPV-associated cervical cancer cells [114]. Early clinical trials have assessed the efficacy of E6/E7 peptide-based HPV therapeutic cancer vaccines [40]. However, those based on VLPs are still at the pre-clinical stage.

Chimeric VLPs have been generated, consisting of the L1 capsid protein plus the entire E7 (11 kDa) or E2 (43 kDa) HPV protein fused to the L2 capsid protein. Efficacy was assessed in a pre-clinical setting using a preventive experimental model. C57BL/6 mice were protected from tumor growth when challenged with the HPV-E7-expressing TC-1 tumor cell line [115].

Similarly, HPV 16 L1-based chimeric virus-like particle (cVLP) were produced in plants containing a string of T cell epitopes from HPV 16 E6 and E7 fused to its C-terminus, and their therapeutic potential and the persistence of IgG neutralizing antibodies were evaluated in a tumor model of C57BL/6 mice [116]. The IgG antibodies persisted for over 12 months and about 57% of tumor reduction was observed.

Although approved L1 HPV-VLPs are prophylactic strategies for preventing HPV infections, as well as subsequent tumor growth, and do not express E7 epitopes, a therapeutic efficacy has been recently demonstrated [117]. Indeed, a meta-analysis showed that the risk of developing recurrent cervical intraepithelial neoplasia is significantly reduced by vaccination with L1 HPV-VLPs after surgical excision [118].

### 4.5. VLPs for Hepatocellular Carcinoma

The clearance of HBsAg in the blood of HBV-positive subjects could be a successful strategy for the treatment of chronic infections that are often not resolved by approved anti-HBV drugs. Indeed, high levels of HBsAg are known to inhibit adaptive, as well as innate, immune response, by leading to tolerance against HBV infection. A vaccine model has been developed to express a 13-mer peptide (SEQ13) from the HBsAg by a novel immuno-enhanced VLP carrier (CR-T3) derived from the round leaf bat HBV core antigen (RBHBcAg). The CR-T3-SEQ13 formulation, by displaying multiple copies of SEQ13, induced a potent antibody response in mice, rabbits, and cynomolgus monkeys, mediating the HBsAg clearance *in vivo* and neutralization of HBV *in vitro* [119].

## 5. VLPs for Delivery of Small Molecule for Cancer Therapy or in Combination with Radiotherapy

VLPs may function as carriers (for delivery to cancer cells) of a high number of molecules, such as proteins, peptides, DNA, RNA, drugs, and imaging substances. An early study showed the expression of a targeting peptide (SP94) in the MS2 VLP to selectively deliver chemotherapeutic drugs (DOX, cisplatin, and 5-FU) to human hepatocellular carcinoma cells (HCC). Such modified VLPs exhibited high avidity and specificity towards HCC with minimal uptake in healthy cells and induced selective cytotoxicity *in vitro*, even at very low doses. For instance, the DOX-loaded VLPs killed HCC cells at IC_50_ values of 10–15 nM, 20 times better than free DOX [120]. Moreover, DOX was encapsulated as well as electrostatically-bound to the exterior of VLPs, based on the red clover necrotic mosaic virus (RCNMV). Different ratios of encapsulated and conjugated DOX regulated the release kinetics of the chemotherapeutic. Indeed, the rapid release of the exterior DOX followed by the slower release of the encapsulated DOX was shown. Qβ VLPs have additionally been investigated with DOX for cancer cell killing. DOX was conjugated using a photocleavable nitroveratryl linker to the exterior of Qβ VLPs that had been modified via a dibromomaleimide chemistry. Light exposure for 15 min caused dose-dependent cytotoxic killing in MCF-7 cells *in vitro* [121]. 

To study the effect of nanoparticle morphology on drug delivery, DOX was loaded on three diverse VLPs: bacteriophage MS2, tobacco mosaic virus (TMV) disks, and filamentous rods of nanophage. The MS2 is spherical in shape with a diameter around 27 nm; the TMV disks are flat and round, measuring 18 × 5 nm, and the nanophages are short filaments, measuring 50 × 6 nm. Intracranial administration of TMV disks and MS2 VLPs in U87Luc glioblastoma-bearing mice determined increased survival rates, with the TMV disk-treated mice showing the greatest efficacy [122]. The nanophage filamentous rods showed no efficacy compared to free DOX and phosphate buffered saline controls, indicating that the carrier itself can also affect drug delivery. 

Other studies have shown that particles with extended surfaces improve drug delivery due to their ability to better interact with vessel walls and to accumulate in tumor tissues. Plant viruses forming filamentous and tubular structures are good candidates for drug delivery platforms. The TMV nanotubes (300 × 18 nm in size) and filamentous PVX (515 × 13 nm) have been successful used to deliver DOX [122]. Moreover, VLPs based on TMV have been used to deliver platin-based drugs, such as cisplatin and phenanthriplatin, to ovarian cancer cells with superior cytotoxicity, and DNA double-strand breakage (DSB) in platinum sensitive and resistant cancer cells compared to free cisplatin [123,124,125]. In addition, TMV–VLPs for targeted delivery of cisplatin have been modified with mannose and lactose moieties, in order to facilitate the interaction between mannose and galectin, as well as lactose and the asialoglycoprotein receptor, which are distributed on the cell membranes. Such modified and drug-loaded TMV–VLPs showed enhanced cytotoxicity in MCF-7 and Hep G2 cancer cell lines [123]. Moreover, VLP derived from the cowpea mosaic virus, produced in plants and injected intratumorally in canine oral melanoma with the magnetic iron oxide hyperthermia nanoparticle (mNPH) as an adjuvant, determined a longer local and distant tumor remission in association with radiotherapy [126].

## 6. VLPs for Cancer Vaccine: Critical Issue

VLPs can be considered as alternatives to traditional vaccines based on their safety, flexibility, and distinctive immunogenic properties, but considerable limitations still persist in their production. For instance, no VLP vaccine currently used in clinical and preclinical trials has induced sterile immunity and total clearance of viral infections, perhaps because most strategies dominantly target one arm of the immune system. 

Production efficiency and scalability are important limits in the production of VLPs. The mammalian cells, which are the only systems to provide an intracellular environment suitable for proper protein folding and post-translational modifications of VLP-forming proteins, have a low production efficacy compared to other VLP systems. In addition, achieving efficient transfection while maintaining cell viability is necessary for successful VLP production. This requires a large number of cells to produce relatively limited amounts of VLPs [78]. Another limitation occurs with the use of peptides as epitope-based vaccines, because they have, in general, limited immunogenicity and need to be administered with appropriate adjuvants or specific antigen delivery systems. 

## 7. Conclusions

Several VLP-based preventive vaccines have been/are currently under development. However, only those targeting HBV and HPV have been approved for human use. All others are still at the pre-clinical stage (Table 4). 

Regarding therapeutic approaches on VLP technology—many are in the preclinical stage and only some are in early stages of human clinical trials. The efficacy of the VLP-based vaccine is highly dependent on the selection of tumor antigens and the design of VLPs. 

Although there are various limitations in the development of the VLP platform, recent advances in the field, the possibility of engineering the VLPs, and the use of appropriate adjuvants are keys that could improve the design and manufacturing of VLPs for prevention and treatment of human cancers.

## Figures and Tables

**Table 1:** Licensed prophylactic VLP vaccine against oncoviruses.

Commercial Name	Virus	Cancer Type	Manufacturers	Tumor Antigens	Adjuvants	Expression System
Engerix-B	HBV	Hepatocellular carcinoma	GSK (UK)	SHBs	Aluminum hydroxide	Yeast: *Saccharomyces cerevisiae*
Recombivax HB (H-B-Vax^®^II)	HBV	Hepatocellular carcinoma	Merck Vaccine (Canada)	SHBs	Aluminum sulfate	Yeast: *Saccharomyces cerevisiae*
Sci-B-Vac^®^ (Bio-Hep-B^®^)	HBV	Hepatocellular carcinoma	SCIgen (Israel Ltd.)	SHBs, MHBs, LHBs	Aluminum hydroxide	Mammalian: CHO cells
Heplisav-B	HBV	Hepatocellular carcinoma	Dynavax (Oakland-USA)	SHBs	1018 ISS	Yeast: *H. polymorpha*
Fendrix	HBV	Hepatocellular carcinoma	GSK (Belgium)	SHBs	AS04 (Aluminum hydroxide and MPL)	Yeast: *Saccharomyces cerevisiae*
Hepavax-Gene	HBV	Hepatocellular carcinoma	Crucell (Dusseldorf-Germany)	SHBs, MHBs	Aluminum hydroxide	Yeast: *H. polymorpha*
Cervarix (bivalent vaccine)	HPV	Cervical cancer, anal cancer, penis cancer, head and neck carcinoma	GSK (UK)	L1 HPV 16 L1 HPV 18	ASO4:Al(OH)_3_ MPL	Baculovirus: Hi-5 baculovirus
Gardasil (quadrivalent vaccine)	HPV	Cervical cancer, anal cancer, penis cancer, head and neck carcinoma	Merck Sharp & Dohme Corp (USA)	L1 HPV6 L1 HPV11 L1 HPV16 L1 HPV18	Amorphous, aluminum, hydroxyphosphate sulfate	Yeast: *Saccharomyces cerevisiae*
Gardasil 9 (nonavalent vaccine)	HPV	Cervical cancer, anal cancer, penis cancer, head and neck carcinoma	Merck Inc. (Canada)	L1 HPV6 L1 HPV11 L1 HPV16 L1 HPV18 L1 HPV31 L1 HPV33 L1 HPV45 L1 HPV52 L1 HPV58	Amorphous, aluminum, hydroxyphosphate sulfate	Yeast: *Saccharomyces cerevisiae*

**Table 2:** Prophylactic VLP vaccine against oncoviruses in preclinical evaluation.

Virus	Cancer Type	Tumor Antigens	Adjuvants	Expression System	References
Human papillomavirus	Cervical cancer	L2 (epitope 20–38)	Montanide	*Pyrococcus furiosus* (PfTrx)	[65]
Human papillomavirus	Cervical cancer	L2 (epitope 17–31)	alum hydroxide	MS2 and PP7	[66]
Human papillomavirus	Cervical cancer	L2 RG1 epitope and VAR2CSA PM antigen	alum hydroxide	AP205 capsid	[75]
Human herpes virus-8	Kaposi’s sarcoma	gpK8.1 ED fused to NDV fusion protein (F)	alum/MPL	Plasmid transfected into CHO or HEK-293 cells	[76]
Epstein–Barr virus	Lymphomas, gastric carcinoma, and nasopharyngeal carcinoma	gp350 and CD21	none	293-VII+ cells or exosomes from HEK293 cells	[72]
Epstein–Barr virus	Lymphomas, gastric carcinoma, and nasopharyngeal carcinoma	gp350/220	none	Plasmid transfected in CHO cells	[26]
Epstein–Barr virus	Lymphomas, gastric carcinoma, and nasopharyngeal carcinoma	EBNA1 and LMP2	none	Plasmid transfected in CHO cells	[77]
Epstein–Barr virus	Lymphomas, gastric carcinoma, and nasopharyngeal carcinoma	gp350, gB, gp42, and gH/gL	aluminum hydroxide and monophosphoryl lipid A	Plasmid transfected in AGS-EBV-eGFP cells	[78]
Epstein–Barr virus	Lymphomas, gastric carcinoma, and nasopharyngeal carcinoma	HBc149, gp350	aluminum hydroxide and AS04	*E. coli*	[79]
HCV Hepatitis C virus	Hepatocellular carcinoma	HBV pre-S1 (20-47)	none	*E. coli*	[80]
HCV Hepatitis C virus	Hepatocellular carcinoma	E1, E2	Alhydrogel	Baculovirus	[81,82]
HCV Hepatitis C virus	Hepatocellular carcinoma	E1, E2	none	Adenovirus	[83]
HBV Hepatitis B virus	Hepatocellular carcinoma	HBsAgS	aluminum hydroxide	Plasmid transfected in human embryonic kidney (HEK) FreeStyle-293F cells	[55]
HBV Hepatitis B virus	Hepatocellular carcinoma	HBc	none	*Pichia pastoris*	[56]

**Table 3:** VLPs for therapeutic vaccine.

Cancer Type	Tumor Antigens	Adjuvants	Expression System	Type of Vaccine	References
Breast cancer	HER2	none	AP205 phage	Preventive and therapeutic	[100]
Breast cancer	IGF-1R	none	Human Parvovirus B19	Preventive and Therapeutic	[101]
Breast cancer	xCT	none	MS2	Therapeutic	[102]
Melanoma	LCMV-TT830–843	Microcrystalline tyrosine (MCT)	CuMVT	Therapeutic	[103]
Melanoma	-	none	Nicotiana bethamiana (CPMV)	Therapeutic	[104]
Melanoma	gp100	none	*E. coli*	Therapeutic	[105]
Pancreatic cancer	mMSLN	none	Baculovirus (SHIV)	Therapeutic	[106]
Pancreatic cancer	hMSLN	none	Baculovirus (SHIV)	Therapeutic	[107]
Hepatocellular carcinoma	CLDN18.2	none	*E. coli*	Therapeutic	[108]

**Table 4:** VLP vaccines in clinical trials.

Cancer Type	Tumor Antigens	Adjuvants	Expression System	Type of Vaccine	Clinical Trial Identifier	Clinical Stage
Vulva and anal cancer	L1	Amorphous, aluminum, hydroxyphosphate sulfate	yeast: *Saccharomyces cerevisiae*	Therapeutic	NCT03051516	Phase IV
HPV infections	L1	Amorphous, aluminum, hydroxyphosphate sulfate	yeast: *Saccharomyces cerevisiae*	Prophylactic	NCT03903562	Phase III
Cervical cancer	L1	Amorphous, aluminum, hydroxyphosphate sulfate	yeast: *Saccharomyces cerevisiae*	Therapeutic	NCT03284866	Phase III
HPV infections	L1	Amorphous, aluminum, hydroxyphosphate sulfate	yeast: *Saccharomyces cerevisiae*	Prophylactic	NCT04235257	Phase IV
Cervical cancer	L1	Amorphous, aluminum, hydroxyphosphate sulfate	yeast: *Saccharomyces cerevisiae*	Therapeutic	NCT00092534	Phase III
Pancreatic cancer	-	none	*Qβbacteriophage*	Therapeutic	NCT04387071	Phase I/II
Melanoma	-	none	*Qβbacteriophage*	Preventive and Therapeutic	NCT04708418	Phase II
Melanoma		Pembrolizumab (anti-PD-1)	*Qβbacteriophage*	Therapeutic	NCT02680184	Phase I

## Data Availability

Data and material are available at https://zenodo.org/deposit/5850860 (accessed on 27 January 2022).
